# Nomogram for predicting the risk of preterm birth in women undergoing in vitro fertilization cycles

**DOI:** 10.1186/s12884-023-05646-x

**Published:** 2023-05-06

**Authors:** Mohan Wang, Mengzhe Hao, Ning Liu, Xiao Yang, Yubin Lu, Ruizhi Liu, Hongguo Zhang

**Affiliations:** Prenatal Diagnosis Center, Reproductive Medicine Center, The First Hospital of Jilin University, Changchun, Jilin China

**Keywords:** Preterm birth, In vitro fertilization, Nomogram, Infertility

## Abstract

**Background:**

The aim of this study was to develop a nomogram for predicting the risk of preterm birth in women undergoing in vitro fertilization (IVF) cycles.

**Methods:**

A retrospective study of 4266 live birth cycles collected from January 2016 to October 2021 at the Center for Reproductive Medicine, First Hospital of Jilin University was performed. The sample size was sufficient based on the minimal ten events per variable (EPV) rule. The primary outcome of this study was preterm birth. The cycles were divided into the preterm birth group (*n* = 827) and the full-term delivery group (*n* = 3439). A nomogram was established based on the multivariate logistic regression analysis results. The area under the curve (AUC) was calculated to assess the prediction accuracy of the nomogram model. The calibration curve was used to measure the calibration of the nomogram.

**Results:**

Multivariate logistic regression analyses showed that female obesity or overweight (*OR* = 1.366, *95% CI*: 1.111–1.679; *OR* = 1.537, *95% CI*: 1.030–2.292), antral follicle count (AFC) of more than 24 (*OR* = 1.378, 95% *CI:* 1.035–1.836), multiple pregnancies (*OR* = 6.748, *95% CI*: 5.559–8.190), gestational hypertension (*OR* = 9.662, *95% CI*: 6.632–14.078) and gestational diabetes (*OR* = 4.650, *95% CI*: 2.289–9.445) were the independent risk factors for preterm birth in IVF patients. The area under curve (AUC) under the receiver operating characteristic (ROC) curve in the prediction model was 0.781(95%CI: 0.763–0.799). The calibration curve of the nomogram showed that the prediction model had a good calibration.

**Conclusions:**

We used five risk factors to conduct a nomogram to predict preterm birth rates for patients undergoing IVF cycles. This nomogram can provide a visual assessment of the risk of preterm birth for clinical consultation.

**Supplementary Information:**

The online version contains supplementary material available at 10.1186/s12884-023-05646-x.

## Background

Assisted reproductive technology (ART) has been more and more widely used. Although ART has contributed to the increase in the live birth rate, the safety problems caused by it should not be ignored. As a complication of ART, preterm birth is one of the leading causes of infant death and neonatal morbidity [1]. Approximately one million babies die yearly due to preterm birth [2]. Related studies of singleton pregnancies have shown that women who underwent in vitro fertilization (IVF) procedures had a higher incidence of preterm birth than those who conceived spontaneously [3–5]. Therefore, the problem of preterm birth of patients assisted by IVF should receive more attention.

Previous studies have demonstrated that preterm birth is associated with many factors, such as maternal demographic characteristics, nutritional status, pregnancy history, adverse behaviors, and so on [6]. It was known that preterm birth was predominantly related to multiple gestation births, regardless of IVF cycles or intrauterine insemination (IUI) cycles [7]. Some researchers thought that the patient's own infertility factors might contribute to the preterm birth rather than the IVF procedures themselves [8]. However, other studies have come to different conclusions. Studies have shown a higher risk of preterm birth after blastocyst embryo transfer than cleavage embryo transfer [9, 10]. Although a sample size study of more than 60,000 showed no significant difference [11].

Researches on risk factors influencing preterm birth in IVF cycles remain inconsistent. Moreover, most studies have looked only at singleton pregnancies. Regarding the safety of the offspring born by ART, it is necessary to predict and intervene the risk factors of preterm birth as early as possible. There is a lack of intuitive and effective methods to assess the risk of preterm birth. This study aimed to establish a nomogram that could predict the risk of preterm birth in IVF cycles and provide early prediction and intervention for preterm birth among Infertile patients.

## Methods

### Study design and subjects

**Fig. 1:**
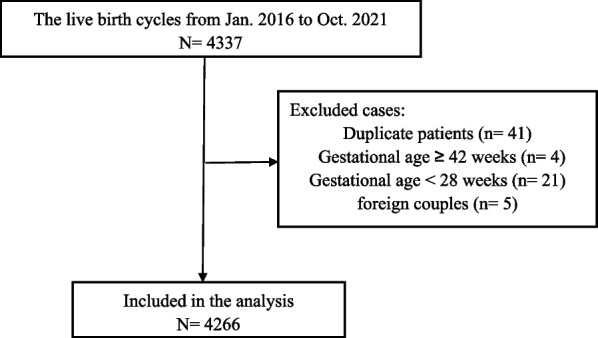
Patient inclusion flowchart

This was a retrospective study, and the data were collected from electronic medical records at the Center for Reproductive Medicine, First Hospital of Jilin University (Changchun, China). Inclusion criteria were patients undergoing IVF cycles with live birth from January 2016 and October 2021. The Sperm donation, preimplantation genetic diagnosis, and preimplantation genetic screening cycles were excluded. Figure 1 shows the patient inclusion process. After exclusions, a total of 4266 cycles were included for analysis. The sample size was evaluated by considering that the reported preterm birth rate was 11.1% [12] and that there were 22 independent variables in this study. 4266 cycles were sufficient based on the minimal 10 events per variable (EPV) rule [13]. The primary outcome of this study was preterm birth. According to the primary outcome, 4266 couples were divided into the preterm birth group and the full-term delivery group, respectively. The study was approved by the ethics committee of the First Hospital of Jilin University (2021–741). Because of the retrospective character of the study, written inform consent was waived by the ethics committee of the First Hospital of Jilin University.

### Definitions and measurements

The variables in the analyses included patients’ sociodemographic characteristics, personal histories, primary diagnosis for infertility, cycle type, chromosome examinations, body mass index (BMI), antral follicle count (AFC), anti-Müllerian hormone (AMH) levels, fertilization method, the thickness of endometrium, embryo transfer stage, number of embryos transferred, and follow-up information of pregnancy. Preterm birth was defined as a live birth with a gestational age between 28 and 37 weeks. Full-term birth was defined as a live birth with a gestational age between 37, not over 42 weeks. BMI was divided into four groups based on the World Health Organization criteria [14]. Based on the patient's personal history recorded, adverse pregnancy history was defined as a history of miscarriage, induced labor, or ectopic pregnancy. The thickness of the endometrium on transfer day was divided into two groups according to a clinical practice guideline for the management of thin endometrium [15].

### Data analysis

SPSS software (Version 27.0) was used to analyse the data. Continuous variables were tested for normality using Kolmogorov–Smirnov tests. Nonnormally distributed data were represented as the median (M) and interquartile range (Q25, Q75). Mann–Whitney U tests were used to investigate group differences. The comparisons of rates between the two groups were performed using the chi-square tests. The significant variables from univariate analyses were put into multivariable logistic regression to determine the independent influencing factors for preterm birth. Statistical package R (version 4.2.2) was used to establish and validate the nomogram. The nomogram was developed based on the results of the multivariate logistic regression analysis and the obtained variables were included in the nomogram to predict the preterm birth rate. The nomogram is based on proportionally converting each regression coefficient in multivariate logistic regression to a scale of 0–100 points. 100 points are assigned to the variable with the highest standardized β coefficient. The points for each independent variable were added together to get the total score for the predicted probability of the preterm birth. The area under the curve (AUC) was calculated to assess the prediction accuracy of the nomogram model. The calibration curve was used to measure the calibration of the nomogram. Statistical significance was set to* p* < 0.05 (two-tailed).

## Results

### Rate of Preterm birth and basic characteristics of patients

A total of 4266 cycles were included in this study. There were 827 cycles in the preterm birth group and 3439 cycles in the full-term birth group. The incidence of preterm birth was 19.39% (827/ 4266). Table 1 presents the basic characteristics of the patients. Female age, male age, female education level, male education level, female BMI, and baseline FSH levels showed differences (*p* < 0.05) in the two groups.

**Table 1:** The basic characteristics of patients with preterm birth and full-term delivery

Variables	Preterm birth	Full-term delivery	*P*
Number of cycles, *n* (%)	827 (19.39)	3439 (80.61)	-
Female age, M (Q_25_, Q_75_)	31 (29, 34)	32 (29, 34)	< 0.001
Male age, M (Q_25_, Q_75_)	32 (30, 35)	33 (30, 36)	< 0.001
Female ethnicity			
Han	752 (90.93)	3096 (90.03)	0.432
Minority	75 (9.07)	343 (9.97)	
Male ethnicity			
Han	757 (91.54)	3128 (90.96)	0.600
Minority	70 (8.46)	311 (9.04)	
Female education level, *n* (%)			
Higher	468 (56.59)	2166 (62.98)	0.002
Secondary	317 (38.33)	1147 (33.35)	
Primary	42 (5.08)	126 (3.67)	
Male education level, *n* (%)			
Higher	464 (56.11)	2112(61.41)	0.019
Secondary	332 (40.14)	1206 (35.07)	
Primary	31 (3.75)	121 (3.52)	
Female BMI, M (Q_25_, Q_75_)	23.00 (20.40, 25.80)	22.21 (20.06, 24.80)	< 0.001
Male BMI, M (Q_25_, Q_75_)^a^	25.39 (22.91, 27.78)	25.35 (22.90, 27.80)	0.964
AFC	22 (15, 24)	20 (13, 24)	< 0.001
Infertility type, *n* (%)			
Primary	548 (66.26)	2251 (65.46)	0.660
Secondary	279 (33.74)	1188 (34.54)	
Infertility duration, M (Q_25_, Q_75_)	4 (2, 6)	3 (2, 5)	0.575
Infertility diagnosis, *n* (%)			
Male factor	303 (36.64)	1339 (54.1)	0.385
Female factor	359 (43.41)	1397 (27.3)	
Male and female factor	163 (19.71)	699 (18.1)	
Unexplained factor	2 (0.24)	4 (0.5)	
Cycle type, *n* (%)			
Fresh	101 (12.21)	504 (14.66)	0.071
Frozen-thaw	726 (87.79)	2935 (85.34)	

The continuous variables are analysed by using Mann Whitney-U test

The categorical variables are analysed by using χ^2^ test

*BMI* Body mass index, *AFC* Antral follicle count

^a^Missing < 1%

### Univariate logistic regression analyses for preterm birth

Univariate logistic regression analyses showed that female age, male age, female education level, male education level, female BMI, AFC, female chromosome, fertilization method, number of embryos transferred, multiple pregnancies, gestational hypertension, and gestational diabetes were associated with preterm birth (*p* < 0.05). (Table 2).

**Table 2:** Univariate logistic regression analyses for preterm birth

Variables	Preterm birth	Full-term delivery	OR	95%CI	*P*
	n(%)	n(%)			
**Female age, years**					0.007
< 35	664 (20.34)	2601 (79.66)	1.000		
≧35, < 40	152 (16.87)	749 (83.13)	0.795	0.655–0.965	0.020
≧40	11 (11.00)	89(89.00)	0.484	0.257–0.911	0.025
**Male age, years**					0.011
< 35	558 (20.64)	2145 (79.36)	1.000		
≧35, < 40	211 (17.93)	966 (82.07)	0.840	0.704–1.001	0.051
≧40	58 (15.03)	328 (84.97)	0.680	0.506–0.912	0.010
**Female ethnicity**					
Han	752 (19.54)	3096 (80.46)	1.000		
Minority	75 (17.94)	343 (82.06)	0.900	0.693–1.170	0.432
**Male ethnicity**					
Han	757 (19.49)	3128 (80.51)	1.000		
Minority	70 (18.37)	311 (81.63)	0.930	0.709–1.220	0.600
**Female education level**					0.002
Higher	468 (17.77)	2166 (82.23)	1.000		
Secondary	317(21.65)	1147 (78.35)	1.279	1.091–1.500	0.002
Primary	42 (25.00)	126 (75.00)	1.543	1.073–2.218	0.019
**Male education level**					0.019
Higher	464 (18.01)	2112 (81.99)	1.000		
Secondary	332 (21.59)	1206 (78.41)	1.253	1.070–1.467	0.005
Primary	31(20.39)	121 (79.61)	1.166	0.776–1.752	0.459
**Female BMI, kg/m**^**2**^					< 0.001
≧18.5, < 25	506 (18.00)	2305 (82.00)	1.000		
< 18.5	60 (15.92)	317 (84.08)	0.862	0.644–1.155	0.320
≧25, < 30	215 (23.81)	688 (76.19)	1.424	1.188–1.706	< 0.001
≧30	46 (26.29)	129 (73.71)	1.624	1.145–2.305	0.007
**AMH, μg/L**^a^					0.056
≥ 2	658 (20.09)	2617 (79.91)	1.000		
≥ 1.0, < 2	82 (16.73)	408 (83.27)	0.799	0.621–1.029	0.082
< 1.0	38 (15.38)	209 (84.62)	0.723	0.507–1.032	0.074
**AFC**					< 0.001
≥ 12, ≤ 24	625 (19.78)	2534 (80.22)	1.000		
< 12	112 (14.74)	648 (85.26)	0.701	0.563–0.872	0.001
> 24	90 (25.94)	257 (74.06)	1.420	1.100–1.833	0.007
**Female chromosome**					0.088
Normal	799 (17.36)	3263 (82.64)	1.000		
Abnormal	24 (13.04)	160 (86.96)	0.613	0.396–0.947	0.028
Not examed	4 (20.00)	16 (80.00)	1.021	0.340–3.062	0.970
**Male chromosome**					0.628
Normal	762 (19.27)	3192 (80.73)	1.000		
Abnormal	60 (21.58)	218 (78.42)	1.153	0.857–1.551	0.347
Not examed	5 (14.71)	29 (85.29)	0.722	0.279–1.872	0.503
**Adverse pregnancy history**					
No	565 (19.37)	2352 (80.63)	1.000		
Yes	262 (19.42)	1087 (80.58)	1.003	0.852–1.181	0.968
**Fertilization method**					
IVF	488 (20.50)	1893 (79.50)			
ICSI	339 (17.98)	1546 (82.02)	0.851	0.729–0.992	0.039
**Cycle type**					
Fresh	101 (16.69)	504 (83.31)	1.000		
Frozen-thaw	726 (19.83)	2935 (80.17)	1.234	0.982–1.551	0.071
**Female smoking**					0.236
Never	807 (19.49)	3333 (80.51)	1.000		
smoking	15 (13.89)	93 (86.11)	0.666	0.384–1.155	0.148
smoked in the past	5 (27.78)	13 (72.22)	1.589	0.565–4.469	0.380
**Male smoking**					0.869
Never	544 (19.17)	2294 (80.83)	1.000		
smoking	277 (19.84)	1119 (80.16)	1.044	0.888 -1.227	0.602
smoked in the past	6 (18.75)	26 (81.25)	0.973	0.399–2.376	0.952
Thickness of endometrium, mm*					
≥ 7 or ≥ 8	803 (19.32)	3353 (80.68)	1.000		
< 7 or < 8	24 (21.82)	86 (78.18)	1.165	0.736–1.844	0.514
**Embryo transfer stage**					
Blastocyst	330 (18.47)	1457 (81.53)	1.000		
Cleavage stage	497 (20.05)	1982 (79.95)	1.107	0.948–1.292	0.197
**Number of embryos transferred**					
1	91 (8.33)	1002 (91.67)	1.000		
2	736 (23.20)	2437 (76.80)	3.325	2.642–4.185	< 0.001
**Multiple pregnancies**					
No	273 (9.25)	2677 (90.75)			
Yes	554 (42.10)	762 (57.90)	7.129	6.040–8.415	< 0.001
**Gestational hypertension**					
No	717 (17.48)	3385 (82.52)	1.000		
Yes	110 (67.07)	54 (32.93)	9.617	6.876–13.451	< 0.001
**Gestational diabetes**					
No	807 (19.11)	3416 (80.89)	1.000		
Yes	20 (46.51)	23 (53.49)	3.681	2.012–6.735	< 0.001

^*^ For fresh IVF-ET cycles, thickness of endometrium was divided into two groups with a 8 mm boundary; for frozen-thaw -ET cycles, thickness of endometrium was divided into two groups with a 7 mm boundary

*BMI* Body mass index, *AMH* Anti-Müllerian hormone, *AFC* Antral follicle count

^a^ Missing < 6%

### Multivariate logistic regression analysis for preterm birth

Table 3 shows the results of the multivariate logistic regression analyses. Female BMI, AFC, fertilization method, multiple pregnancies, gestational hypertension, and gestational diabetes were independent influencing factors for preterm birth (*p* < 0.05). Female patients with a BMI of at least 25 and less than 30 had increased odds of preterm births compared to those in the normal BMI range (*OR* = 1.366, *95% CI*: 1.111–1.679). Female patients with a BMI of 30 or more were also more likely to have preterm births than those in the normal BMI range (*OR* = 1.537, *95% CI*: 1.030–2.292). Female patients with AFC of more than 24 had increased odds of preterm births compared to those with AFC between 12 and 24 (*OR* = 1.378, 95% *CI:* 1.035–1.836). Multiple pregnancies increased the odds of preterm birth compared to singleton pregnancy (*OR* = 6.744, *95% CI*: 5.556–8.186). Moreover, gestational hypertension and gestational diabetes were significantly associated with preterm birth, respectively (*OR* = 9.662, *95% CI*: 6.632–14.078; *OR* = 4.650, *95% CI*: 2.289–9.445). Furthermore, we performed stratified analyses based on the number of pregnancies. For singleton pregnancies, female underweight or obesity (*OR* = 0.509, *95% CI*: 0.269–0.963; *OR* = 1.771, *95% CI*: 1.328–2.363), gestational hypertension (*OR* = 11.512, *95% CI*: 7.211–18.376) and gestational diabetes (*OR* = 3.785, *95% CI*: 1.531–9.358) were associated with preterm birth. For multiple pregnancies, female overweight (*OR* = 2.402, *95% CI*: 1.146–3.639), AFC of more than 24 (*OR* = 1.543, 95% *CI:* 1.052–2.263), gestational hypertension (*OR* = 7.129, *95% CI*: 4.016–12.653) and gestational diabetes (*OR* = 6.431, *95% CI*: 1.779–23.241) were associated with preterm birth. (See Supplementary Table 2, Additional File 1).

**Table 3:** Multivariate logistic regression analysis for preterm birth

Variables	OR	95%CI	*P*
**Female BMI, kg/m**^**2**^			0.002
≧18.5, < 25	1.000		
< 18.5	0.827	0.597–1.145	0.252
≧25, < 30	1.366	1.111–1.679	0.003
≧30	1.537	1.030–2.292	0.035
**Number of AFC**			0.034
≥ 12, ≤ 24	1.000		
< 12	0.871	0.680–1.115	0.273
> 24	1.378	1.035–1.836	0.028
**Multiple pregnancies**			
No	1.000		
Yes	6.744	5.556–8.186	< 0.001
**Gestational hypertension**			
No	1.000		
Yes	9.662	6.632–14.078	< 0.001
**Gestational diabetes**			
No	1.000		
Yes	4.650	2.289–9.445	< 0.001

*BMI* Body mass index, *AFC* Antral follicle count

### Development and validation of the nomogram

**Fig. 2:**
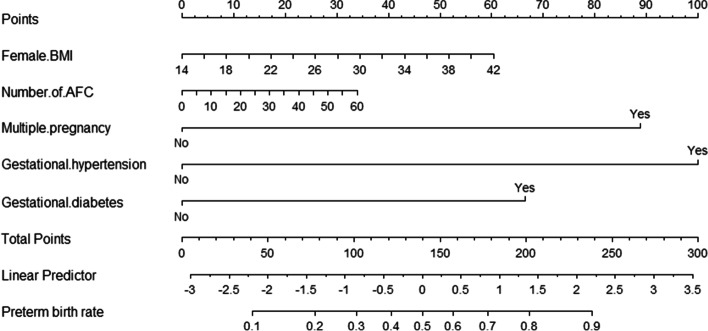
Nomogram for predicting the risk of preterm birth. *BMI *Body mass index, *AFC* Antral follicle count. Example prediction nomogram for risk of preterm birth. A couple with IVF treatment: female BMI = 30 kg/m^2^ (34 points), number of AFC = 10 (6 points), singleton pregnancy (0 points), no gestational hypertension (0 points), gestational diabetes (67 points). The total point reaches 107, with an estimated probability of preterm birth was 0.32 (32%)

**Fig. 3:**
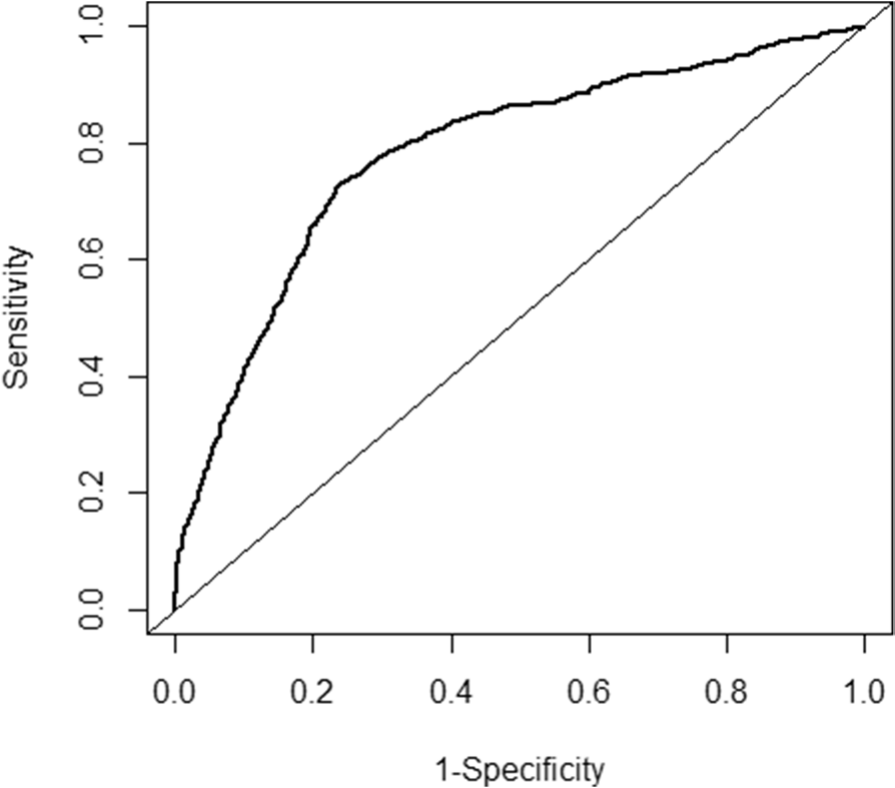
Receiver operating characteristic (ROC) curve of the nomogram

**Fig. 4:**
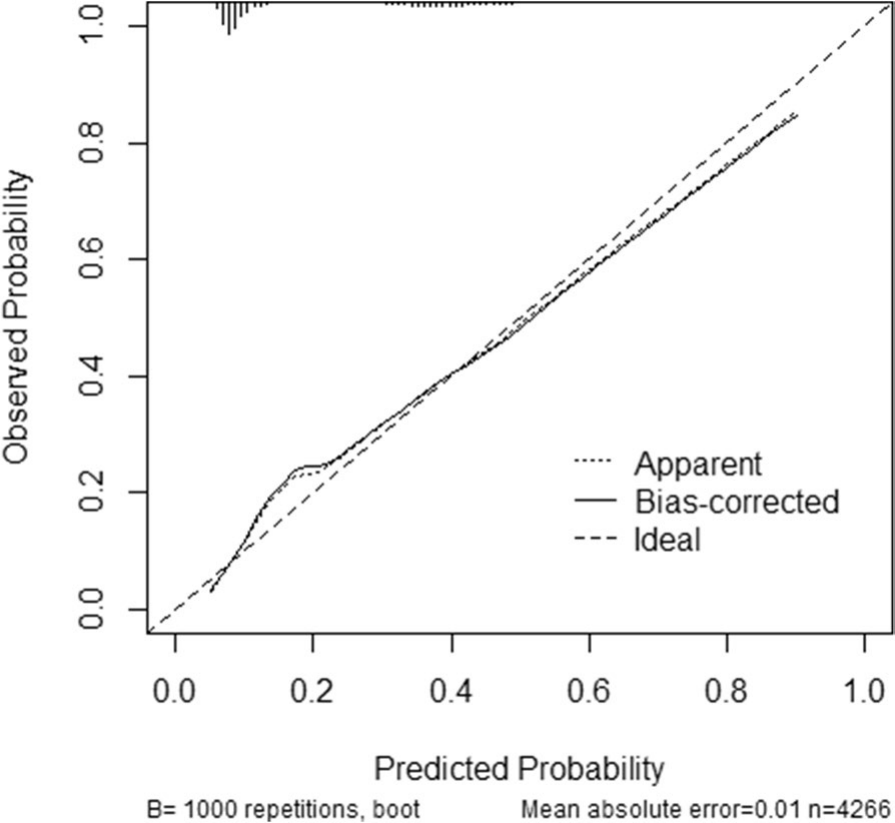
Calibration curves of the nomogram

Based on the multivariate logistic regression analyses, the five independent factors were included to establish the nomogram for predicting the risk of preterm birth (Fig. 2). The receiver operating characteristic (ROC) curve showed that our model exhibited a relatively good prediction capability, with an AUC of 0.781 ((95%CI: 0.763–0.799; Fig. 3). The calibration curve of the nomogram shows that the prediction model had a good calibration. (Fig. 4).

## Discussion

There is a lack of studies using nomograms to predict the risk of preterm birth in IVF cycles. This nomogram we developed aimed to predict the individual probability of preterm birth for infertility patients undergoing IVF cycles intuitively. The nomogram showed that the predictors of preterm birth in our model were female BMI, AFC, multiple pregnancies, gestational hypertension, and gestational diabetes.

BMI is a known influencing factor for preterm birth. Our results suggested that obesity and overweight increased the odds of preterm birth compared to patients with normal BMI. These were consistent with previous findings [16, 17]. Obese or overweight women are more likely to conceive fetuses with birth defects such as neural tube defects, and these infants are more prone to be born prematurely [18]. Women with BMIs outside the normal range are also more likely to develop diabetes. In this study, another risk factor for preterm birth in IVF-assisted pregnancies was gestational diabetes. Gestational diabetes affects about six percent of pregnant women, and the prevalence is increasing with obesity [19]. About three-quarters of patients with gestational diabetes can be managed with appropriate physical activity, diets, and lifestyle changes [19]. This could also promote weight loss in obese or overweight patients. By using the nomogram of this study, we can predict the rate of preterm birth that will decrease with the reduction of female BMI and set feasible targets for IVF patients.

AFC can be used as a valuable parameter for predicting ovarian response and pregnancy during ART cycles [20]. There was no evidence that abnormal AFC was an independent predictor of preterm birth. Our findings suggested that excessive antral follicles were associated with increased odds of preterm birth in IVF cycles. A high AFC usually indicates the possibility of the polycystic ovarian syndrome (PCOS) [21]. Studies indicated that PCOS increased the risk of a twofold higher chance of preterm birth [22, 23]. This might partly explain why AFC could be a predictor of preterm birth. Like most studies, this study also demonstrated that multiple pregnancies were a strong risk factor for preterm birth, regardless of whether the subjects were ART-assisted pregnancy patients [24, 25]. We concluded that patients with multiple pregnancies during IVF cycles were 6.744 times more likely to have preterm births than single pregnancies. Single embryo transfer is usually recommended for ART [26]. This is the most effective way to reduce the rate of multiple pregnancies. Although the number of embryos transferred was not significantly associated with preterm birth in this study, we still need to reduce the multiple pregnancy rate by controlling the number of transferred embryos or performing embryo reductions to optimize birth outcomes.

Another strong risk factor in this article was gestational hypertension. It is one of the most common pregnancy complications and causes of maternal morbidity and mortality. A meta-analysis confirmed that ART pregnancies had higher odds of gestational hypertension than spontaneous pregnancies [27]. The mechanism of gestational hypertension in IVF cycles remains to be investigated. A large study of nearly 600,000 mothers with ART showed that multiple pregnancies were the single most likely explanation for the increased rate of gestational hypertension [28]. Given the current findings, there is still a need to advocate for single embryo transfer to see if it can reduce the risk of gestational hypertension for IVF patients.

To the best of our knowledge, this was the first nomogram prediction model for predicting the risk of preterm birth in patients undergoing IVF cycles. We visualized the prediction model by nomogram to make it easier to understand. The points corresponding to each risk factor were obtained in the nomogram. Total points were obtained by adding five points together. The preterm birth rate based on the total points was each patient's probability of preterm birth. Furthermore, two of our five predictors were maternal factors that could predict preterm birth risk before IVF treatment. And for those predicting factors that arise during pregnancies, timely monitoring should be conducted to reduce the probability of occurrence of risk factor events to reduce the risk of preterm birth. However, several limitations of this study should be noted. First, this is a retrospective study, and the evidence is less strong than in prospective studies. Secondly, this single-center study brings about selection bias and requires further validation with data from multiple centers for external verification. Moreover, our variables came from the electronic medical records, and other relevant variables, such as the causes of preterm birth, needed to be adequately collected. Therefore, we could not stratify spontaneous and therapeutic preterm births, although there would be differences in risk factors for these two subtypes of preterm birth. Finally, the subgroup of early preterm births was not analysed, and there may be different predictors for such patients.

## Conclusions

We used the female BMI, AFC, multiple pregnancy, gestational hypertension, and gestational diabetes to conduct a nomogram for predicting preterm birth rates for patients undergoing IVF cycles. This nomogram can provide a visual assessment of the risk of preterm birth, clinical consultation, and preventive advice for patients before they enter the cycles of IVF treatment.

## Supplementary Information

**Additional file 1.**

## Data Availability

The datasets analysed during the current study are not publicly available due to the hospital policy and patients’ privacy, but are available from the corresponding author on reasonable request.

## Abbreviations

IVF: In vitro fertilization
AUC: Area under curve
ROC: Receiver operating characteristic
ART: Assisted reproductive technology
IUI: Intrauterine insemination
EPV: Events per variable
BMI: Body mass index
AFC: Antral follicle count
AMH: Anti-Müllerian hormone
PCOS: Polycystic ovarian syndrome
