# Supplementary material for: Nomogram for predicting the risk of preterm birth in women undergoing in vitro fertilization cycles
Source: BMC Pregnancy Childbirth. 2023 May 6;23:324. doi: 10.1186/s12884-023-05646-x (PMC10163771; doi:10.1186/s12884-023-05646-x)
Supplement: Supplementary file 1 — Additional file 1. [file 12884_2023_5646_MOESM1_ESM.docx]

**Supplementary Table 1. Univariate logistic regression analyses of preterm births for singleton and multiple pregnancies.**

| **Variables** | **Singleton pregnancy (N=2950)** | | | | **Multiple pregnancies (N=1366)** | | | | |
| --- | --- | --- | --- | --- | --- | --- | --- | --- | --- |
|  | **Preterm birth**  **n (%)** | **OR** | **95%CI** | ***P*** | **Preterm birth**  **n (%)** | **OR** | **95%CI** | ***P*** |  |
| **Female age, years** |  |  |  | 0.332 |  |  |  | 0.167 |  |
| <35 | 190 (8.81) | 1.000 |  |  | 474 (42.74) | 1.000 |  |  |  |
| ≧35, <40 | 76 (10.66) | 1.235 | 0.932-1.634 | 0.141 | 76 (40.43) | 0.909 | 0.664-1.245 | 0.553 |  |
| ≧40 | 7 (8.64) | 0.979 | 0.445-2.155 | 0.958 | 4 (21.05) | 0.357 | 0.118-1.083 | 0.069 |  |
| **Male age, years** |  |  |  | 0.470 |  |  |  | 0.113 |  |
| <35 | 156 (8.73) | 1.000 |  |  | 402 (43.89) | 1.000 |  |  |  |
| ≧35, <40 | 85 (9.98) | 1.159 | 0.878-1.530 | 0.299 | 126 (38.77) | 0.810 | 0.625-1.048 | 0.109 |  |
| ≧40 | 32 (10.29) | 1.199 | 0.803-1.791 | 0.375 | 26 (34.67) | 0.678 | 0.414-1.111 | 0.123 |  |
| **Female ethnicity** |  |  |  |  |  |  |  |  |  |
| Han | 242 (9.11) | 1.000 |  |  | 510 (42.75) | 1.000 |  |  |  |
| Minority | 31 (10.51) | 1.171 | 0.789-1.738 | 0.434 | 44 (35.77) | 0.746 | 0.507-1.097 | 0.137 |  |
| **Male ethnicity** |  |  |  |  |  |  |  |  |  |
| Han | 247 (9.21) | 1.000 |  |  | 510 42.39) | 1.000 |  |  |  |
| Minority | 26 (9.70) | 1.059 | 0.692-1.620 | 0.791 | 44 (38.94) | 0.866 | 0.584-1.286 | 0.477 |  |
| **Female education level** |  |  |  | 0.157 |  |  |  | 0.001 |  |
| Higher | 162 (8.85) | 1.000 |  |  | 306 (38.11) | 1.000 |  |  |  |
| Secondary | 95 (9.43) | 1.717 | 0.987-2.986 | 0.055 | 222 (48.58) | 1.408 | 0.817-2.425 | 0.218 |  |
| Primary | 16 (14.29) | 1.073 | 0.823-1.400 | 0.603 | 26 (46.43) | 1.534 | 1.216-1.935 | <0.001 |  |
| **Male education level** |  |  |  | 0.145 |  |  |  | 0.095 |  |
| Higher | 151 (8.44) | 1.000 |  |  | 313 (39.77) | 1.000 |  |  |  |
| Secondary | 112 (10.66) | 1.085 | 0.554-2.122 | 0.812 | 220 (45.17) | 1.514 | 0.813-2.819 | 1.191 |  |
| Primary | 10 (9.09) | 1.294 | 1.000-1.673 | 0.050 | 21 (50.00) | 1.248 | 0.993-1.568 | 0.058 |  |
| **Female BMI, kg/m^2^** |  |  |  | <0.001 |  |  |  | 0.047 |  |
| ≧18.5, <25 | 160 (8.18) | 1.000 |  |  | 346 (40.47) | 1.000 |  |  |  |
| <18.5 | 11 (4.35) | 0.510 | 0.273-0.954 | 0.035 | 49 (39.52) | 0.961 | 0.654-1.413 | 0.840 |  |
| ≧25, <30 | 87 (14.05) | 1.836 | 1.390-2.425 | <0.001 | 128 (45.07) | 1.207 | 0.921-1.582 | 0.173 |  |
| ≧30 | 15 (12.30) | 1.574 | 0.895-2.766 | 0.115 | 31 (58.49) | 2.073 | 1.180-3.641 | 0.011 |  |
| **AMH,μg/L** |  |  |  | 0.935 |  |  |  | 0.712 |  |
| ≥2 | 201 (9.13) | 1.000 |  |  | 457 (42.59) | 1.000 |  |  |  |
| ≥1.0, <2 | 36 (9.70) | 1.070 | 0.737-1.554 | 0.723 | 46 (38.66) | 0.849 | 0.576-1.252 | 0.410 |  |
| <1.0 | 19 (9.41) | 1.034 | 0.631-1.694 | 0.896 | 19 (42.22) | 0.985 | 0.539-1.802 | 0.961 |  |
| **Number of AFC** |  |  |  | 0.198 |  |  |  | 0.071 |  |
| ≥12, ≤24 | 199 (9.32) | 1.000 |  |  | 426 (41.64) | 1.000 |  |  |  |
| <12 | 47 (7.97) | 0.843 | 0.605-1.174 | 0.311 | 65 (38.24) | 0.868 | 0.622-1.211 | 0.403 |  |
| >24 | 27 (12.05) | 1.334 | 0.870-2.046 | 0.187 | 63 (51.22) | 1.471 | 1.012-2.140 | 0.043 |  |
| **Female chromosome** |  |  |  | 0.480 |  |  |  | 0.199 |  |
| Normal | 261 (9.32) | 1.000 |  |  | 538 (42.60) | 1.000 |  |  |  |
| Abnormal | 10 (7.19) | 0.754 | 0.391-1.452 | 0.398 | 14 (31.11) | 0.609 | 0.321-1.155 | 0.129 |  |
| Not examed | 2 (16.67) | 1.945 | 0.424-8.924 | 0.392 | 2 (25.00) | 0.449 | 0.090-2.234 | 0.328 |  |
| **Male chromosome** |  |  |  | 0.915 |  |  |  | 0.277 |  |
| Normal | 253 (9.25) | 1.000 |  |  | 509 (41.76) | 1.000 |  |  |  |
| Abnormal | 20 (10.15) | 1.109 | 0.686-1.792 | 0.674 | 40(49.38) | 1.361 | 0.868-2.135 | 0.18 |  |
| Not examed | 0 (0.00) | 0.000 | - | 0.998 | 5 (31.25) | 0.634 | 0.219-1.836 | 0.401 |  |
| **Adverse pregnancy history** |  |  |  |  |  |  |  |  |  |
| No | 177 (8.82) | 1.000 |  |  | 388 (42.64) | 1.000 |  |  |  |
| Yes | 96 (10.18) | 1.172 | 0.902-1.522 | 0.235 | 166 (40.89) | 0.931 | 0.734-1.180 | 0.552 |  |
| **Fertilization method** |  |  |  |  |  |  |  |  |  |
| IVF | 163 (9.99) | 1.000 |  |  | 325 (43.33) | 1.000 |  |  |  |
| ICSI | 110 (8.34) | 0.819 | 0.636-1.056 | 0.124 | 229 (40.46) | 0.889 | 0.712-1.109 | 0.296 |  |
| **Cycle type** |  |  |  |  |  |  |  |  |  |
| Fresh | 34 (7.98) | 1.000 |  |  | 67 (37.43) | 1.000 |  |  |  |
| Frozen-thaw | 239 (9.47) | 1.026 | 0.829-1.754 | 0.328 | 487 (42.83) | 1.252 | 0.905-1.733 | 0.174 |  |
| **Female smoking** |  |  |  | 0.998 |  |  |  | 0.812 |  |
| Never | 272 (9.47) | 1.000 |  |  | 535 (42.16) | 1.000 |  |  |  |
| smoking | 0 (0.00) | 0.000 | - | 0.997 | 15 (38.46) | 0.857 | 0.446-1.650 | 0.645 |  |
| smoked in the past | 1 (10.00) | 1.062 | 0.134-8.412 | 0.955 | 4 (50.00) | 1.372 | 0.342-5.510 | 0.656 |  |
| **Male smoking** |  |  |  | 0.799 |  |  |  | 0.626 |  |
| Never | 175(9.00) | 1.000 |  |  | 369 (41.32) | 1.000 |  |  |  |
| smoking | 96 (9.76) | 1.093 | 0.842-1.420 | 0.503 | 181 (43.93) | 1.113 | 0.879-1.409 | 0.375 |  |
| smoked in the past | 2 (9.52) | 1.065 | 0.246-4.609 | 0.952 | 4 (36.36) | 0.811 | 0.236-2.792 | 0.740 |  |
| **Thickness of endometrium, mm** |  |  |  |  |  |  |  |  |  |
| ≥ 7 or≥8 | 265 (9.22) | 1.000 |  |  | 538 (42.00) | 1.000 |  |  |  |
| < 7 or<8 | 8 (10.67) | 1.176 | 0.559-2.475 | 0.669 | 16 (45.71) | 1.163 | 0.593-2.282 | 0.661 |  |
| **Embryo transfer stage** |  |  |  |  |  |  |  |  |  |
| Blastocyst | 119 (9.08) | 1.000 |  |  | 211 (44.33) | 1.000 |  |  |  |
| Cleavage stage | 154 (9.40) | 1.039 | 0.808-1.335 | 0.766 | 343 (40.83) | 0.867 | 0.694-1.088 | 0.217 |  |
| **Number of embryos transferred** |  |  |  |  |  |  |  |  |  |
| 1 | 83 (7.71) | 1.000 |  |  | 8 (47.06) | 1.000 |  |  |  |
| 2 | 190 (10.14) | 1.350 | 1.031-1.767 | 0.029 | 546 (42.03) | 0.816 | 0.313-2.128 | 0.677 |  |
| **Gestational hypertension** |  |  |  |  |  |  |  |  |  |
| No | 232 (8.08) | 1.000 |  |  | 485 (39.37) | 1.000 |  |  |  |
| Yes | 41 (51.25) | 11.954 | 7.558-18.907 | <0.001 | 69 (82.14) | 7.085 | 4.007-12.527 | <0.001 |  |
| **Gestational diabetes** |  |  |  |  |  |  |  |  |  |
| No | 266 (9.10) | 1.000 |  |  | 541 (41.62) | 1.000 |  |  |  |
| Yes | 7 (25.93) | 3.496 | 1.465-8.344 | 0.005 | 13 (81.25) | 6.079 | 1.724-21.437 | 0.005 |  |

**Supplementary Table 2. Multivariate logistic regression analyses of preterm births for singleton and multiple pregnancies.**

| **Variables** | **Singleton pregnancy (N=2950)** | | | **Multiple pregnancies (N=1366)** | | |
| --- | --- | --- | --- | --- | --- | --- |
|  | **OR** | **95%CI** | ***P*** | **OR** | **95%CI** | ***P*** |
| Female BMI, kg/m2 |  |  | <0.001 |  |  | 0.104 |
| ≧18.5, <25 | 1.000 |  |  | 1.000 |  |  |
| <18.5 | 0.509 | 0.269-0.963 | 0.038 | 1.001 | 0.672-1.490 | 0.998 |
| ≧25, <30 | 1.771 | 1.328-2.363 | <0.001 | 1.120 | 0.845-1.486 | 0.430 |
| ≧30 | 1.260 | 0.685-2.318 | 0.457 | 2.042 | 1.146-3.639 | 0.015 |
| **Number of AFC** |  |  | -- |  |  | 0.052 |
| ≥12, ≤24 | -- |  | -- | 1.000 |  |  |
| <12 | -- | -- | -- | 0.882 | 0.624-1.247 | 0.478 |
| >24 | -- | -- | -- | 1.543 | 1.052-2.263 | 0.026 |
| **Gestational hypertension** |  |  |  |  |  |  |
| No | 1.000 |  |  | 1.000 |  |  |
| Yes | 11.512 | 7.211-18.376 | <0.001 | 7.129 | 4.016-12.653 | <0.001 |
| **Gestational diabetes** |  |  |  |  |  |  |
| No | 1.000 |  |  | 1.000 |  |  |
| Yes | 3.785 | 1.531-9.358 | 0.004 | 6.431 | 1.779-23.241 | 0.005 |


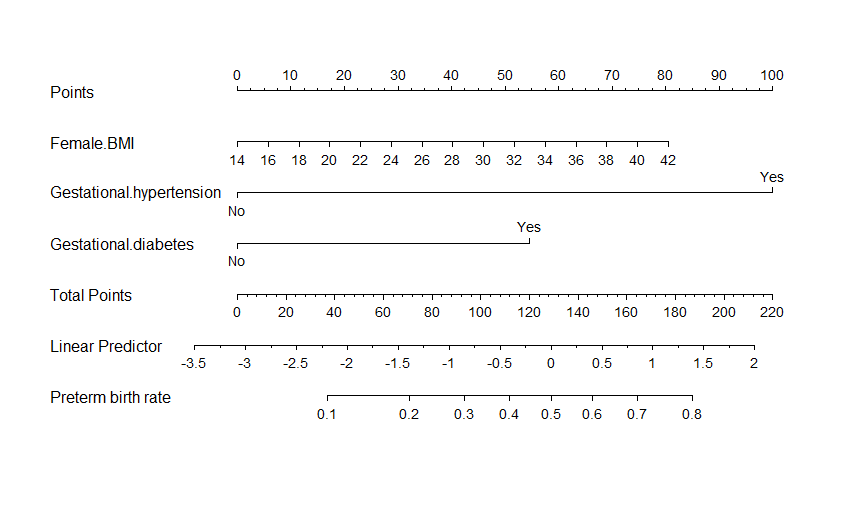


**Supplementary Figure 1. Nomogram for predicting the risk of preterm birth in singleton pregnancy.**


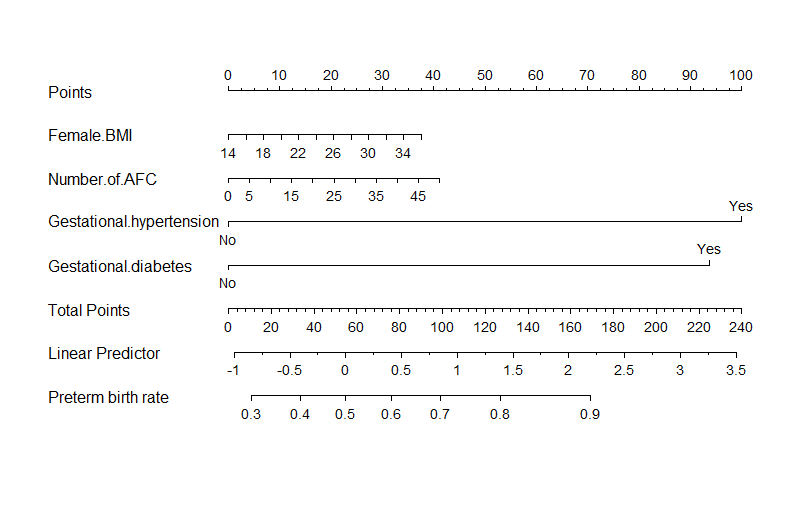


**Supplementary Figure 2. Nomogram for predicting the risk of preterm birth in multiple pregnancies.**
